# Induction Chemotherapy Plus Simultaneous Modulated Accelerated Radiation Therapy in Non-operative Hypopharyngeal and Supraglottic Laryngeal Squamous Cell Carcinoma: Long-Term Outcome of a Prospective Phase 2 Study

**DOI:** 10.3389/fonc.2021.637978

**Published:** 2021-03-12

**Authors:** Boning Cai, Lingling Meng, Jingzi Mo, Shouping Xu, Baolin Qu, Fang Liu, Lin Ma

**Affiliations:** ^1^Department of Radiation Oncology, The First Medical Center, Chinese PLA General Hospital, Beijing, China; ^2^Department of Hematology and Oncology, The 921st Hospital of Chinese PLA, The Second Affiliated Hospital of Hunan Normal University, Changsha, China

**Keywords:** hypopharynx, laryngeal neoplasms, squamous cell carcinoma, intensity-modulated radiotherapy, chemoradiotherapy

## Abstract

**Background:** To evaluate the toxicities and long-term outcomes of induction chemotherapy (ICT) plus simultaneous modulated accelerated radiation therapy (SMART) in non-operative hypopharyngeal and supraglottic laryngeal squamous cell carcinoma (SCCH/L).

**Materials and Methods:** This was a prospective phase 2 study. Patients diagnosed with SCCH/L, aged from 18 to 75, staged from III to IVB in accordance with the AJCC 2010 criteria, and refusing surgery were eligible. The patients were treated with 2–3 cycles of docetaxel-cisplatin-based ICT and SMART combined with 2–3 cycles of cisplatin-based concurrent chemotherapy. The prescription dose to the primary tumor and metastatic nodes was 69 Gy in 30 fractions. Acute and late toxicities were assessed according to the established Radiation Therapy Oncology Group/European Organization for Research and Treatment of Cancer (RTOG/EORTC) criteria, and long-term outcomes were analyzed.

**Results:** Between February 2013 and June 2015, 55 newly diagnosed SCCH/L patients were enrolled. No grade 2 or worse acute xerostomia was noted. The incidences of grade 3 acute dermatitis, oral mucositis, and pharyngoesophagitis were 12.7, 3.6, and 12.7%, respectively. The median follow-up time was 48 months (range 5.5–74 months). The main late toxicity was hoarseness or sore throat, with an incidence of 32.7%. The 5-year functional larynx-preservation survival was 51.5%. The 3- and 5-year locoregional control and overall survival were 58.2, 51.5, 63.6, and 54.1%, respectively.

**Conclusions:** The ICT plus SMART with a regimen of 69 Gy/30 F for the treatment of SCCH/L demonstrated acceptable severe toxicity, satisfactory long-term outcomes, and laryngeal function preservation.

## Background

The complicated anatomical structures of locally advanced squamous cell carcinoma of the hypopharynx and supraglottic larynx (LA-SCCH/L) and the goal of laryngeal function retention dictate that radiation therapy and chemotherapy are the primary conservative treatments for LA-SCCH/L, ensuring the efficacy while retaining the deglutition and phonation functions (1). Compared with chemotherapy or radiation therapy alone, concurrent chemoradiotherapy (CCRT) is more effective in terms of local control and distant metastasis reduction and improves survival (2, 3); thus, it has become the main treatment for the non-operable LA-SCCH/L (4, 5). The common modality is the cisplatin-based CCRT with or without induction chemotherapy (ICT) (6, 7).

For LA-SCCH/L, the treatment efficacy, toxicities, and laryngeal function preservation are related to the irradiation dose (8, 9). The intensity-modulated radiation therapy (IMRT) can deliver a highly conformed dose to targets while effectively sparing critical organs. It has the potential to improve the local control rate and reduce radiation-related toxicities, and its unique technique allows for variable doses to be delivered to different targets simultaneously (10). The local control rate could be improved by increasing the fractional dose to the tumor bed, and the overall treatment time could be shortened to reduce the postprocedure-accelerated repopulation of tumor cells. However, the best regimen in terms of efficacy, organ preservation, and acceptable toxicity remains to be determined (11, 12).

In our previous study, simultaneous modulated accelerated radiation therapy (SMART) showed minor acute severe toxicities and led to satisfactory short-term outcomes in patients with non-operative LA-SCCH/L (13). In this paper, the long-term outcome will be evaluated.

## Materials and Methods

### Patients and Trial Design

This study was a single-center, prospective phase 2 study, which is registered with ChiCTR-ONRC-14004240, and began enrolling patients in February 2013. Patients newly diagnosed with LA-SCCH/L, aged from 18 to 75, with III to IVA stage tumors and who refused surgery, or stage IVB tumors were eligible. The clinical stage was determined according to the American Joint Committee on Cancer 2010 criteria. The included patients had normal routine blood tests and hepatorenal function and did not have any severe and/or uncontrolled medical conditions, including severe cardiovascular disease, uncontrolled diabetes and hypertension, active infection, and liver and kidney disorders. Patients who received any surgical operation or radiotherapy or documented hypersensitivity reaction to paclitaxel or docetaxel were excluded. Patients were fully evaluated with PET-CT or MRI of the head and neck, chest CT, barium esophagography or panendoscopy, abdominal ultrasound, and bone scans to be identified without a second primary tumor. The Karnofsky scores were from 80 to 100. As reviewed by the Ethics Committee of the Chinese People's Liberation Army (PLA) General Hospital, patients who started treatment in our center after the enrollment were recommended to participate in this single-arm prospective study.

### Radiation Therapy

The gross target volume (GTV) of the primary tumor and metastatic lymph nodes were both defined as grossly visible primary tumor and metastatic lymphadenopathy on CT or MRI images. The planning target volume of the primary tumor and metastatic lymph node (pGTV) was obtained by expanding the corresponding GTV with a margin of 3 mm. The clinical target volume (CTV) included high-risk (CTV1) and low-risk volumes (CTV2). Each CTV was automatically expanded to generate the corresponding planning target volume (PTV) with an isotropic 3-mm margin and at least 3 mm from the skin surface. The organs at risk (OARs), including the parotid glands, oral cavity, spinal cord, and esophagus–trachea (E–T, ranging from annular cartilage to 1 cm below to PTV2), were also delineated. The prescription doses to pGTV, PTV1, and PTV2 were 69, 60, and 54 Gy, respectively, in 30 fractions. With an α/β value of the tumor defined as 10 Gy, the biologically effective dose (BED) of pGTV was 84.70 Gy. The dose–volume planning constraints for OARs in our center (14): spinal cord maximum dose (Dmax) < 45 Gy, oral cavity V_40_ < 30%, parotid mean dose (Dmean) < 28 Gy, and E–T V_40_ < 30%.

### Induction Chemotherapy and CCRT

Patients were treated with two–three cycles of docetaxel–cisplatin (TP)-based ICT followed by cisplatin-based CCRT (14). The TP regimen included docetaxel 70 mg/m^2^ on day 1 and cisplatin 40 mg/m^2^ on days 1 and 2 every 3 weeks. Cisplatin at a dose of 80 mg/m^2^ was delivered on days 1 and 22 of radiotherapy. Every patient was treated with at least one cycle of concurrent chemotherapy, and the third cycle was delivered on day 43 of radiotherapy if possible.

### Clinical Evaluation and Follow-Up

Acute and late toxicities were defined and graded according to the established Radiation Therapy Oncology Group/European Organization for Research and Treatment of Cancer (RTOG/EORTC) criteria (15). Acute toxicities were evaluated weekly, and peak toxicities were recorded. The treatment response was primarily evaluated 1 month after radiation therapy with the Response Evaluation Criteria in Solid Tumors (RECIST) Version 1.1 (RECIST Working Group, 2009) based on MRI. Follow-up examinations were conducted every 3 months for years 1 and 2, every 6 months for years 3–5, and then annually thereafter.

### Statistical Analysis

All data analyses were performed using SPSS 19.0 software package (IBM Inc., United States). The Pearson's chi-square test was used for the bivariate analysis, and the Mann–Whitney test or Kruskal–Wallis rank test was used for determining continuous variables. The laryngeal function preservation was defined as no tracheotomy or gastric tube diet along with no local recurrence of the primary tumor. The 3- and 5-year locoregional control (LRC) and overall survival (OS) were estimated using the Kaplan–Meier method. The Cox regression analysis was adopted for the multiple-factor analysis of survival. Values of *p* < 0.05 were considered statistically significant.

Because our previous study did not find an effect of tumor burden on survival (13), the secondary subgroup survival analysis was carried out on the following four staging cohorts [based on National Comprehensive Cancer Network treatment recommendations and drawn on the study of Patel et al. (16)]: (1) T2–T3N0–N1 (non-T4, low nodal burden group), (2) T2–T3N2 (non-T4, high nodal burden group), (3) T4aN-any (T4, high tumor burden group), and (4) T4bN-any (very advanced group).

## Results

Between February 2013 and June 2015, 55 patients with newly diagnosed locally advanced squamous cell carcinoma of the hypopharynx (*n* = 42) and supraglottic larynx (*n* = 13) were enrolled. The patients ranged in age from 42 to 73 years, with a mean age of 57.64 years. Patient characteristics have been previously described and are summarized in Table 1.

**Table 1:** Patient characteristics (*n* = 55).

**Characteristics**		**Value (%)**
Age (years)	Median (range)	57.64 (42–73)
Sex	Male	54 (98.2)
	Female	1 (1.8)
Tumor site	Hypopharyngeal	42 (76.4)
	Supraglottic	13 (23.6)
Tumor classification	T1-2	15 (27.2)
	T3	16 (29.1)
	T4a	14 (25.5)
	T4b	10 (18.2)
Lymph node status	N0-1	11 (20.0)
	N2b	20 (36.4)
	N2c	24 (43.6)
Clinical stage	III	9 (16.4)
	IVA	36 (65.5)
	IVB	10 (18.2)
Tumor burden	T2-T3N0-N1 (non-T4, low nodal burden group)	8 (14.5)
	T1-T3N2 (non-T4, high nodal burden group)	23 (41.8)
	T4aN-any (T4, high tumor burden group)	14 (25.5)
	T4bN-any (very advanced group)	10 (18.2)
ICT cycles	2	50 (90.9)
	3	5 (9.1)
CCRT cycles	1	2 (3.6)
	2	44 (80.0)
	3	9 (16.4)

*ICT, induction chemotherapy; CCRT, concurrent chemoradiotherapy*.

Three IMRT techniques were applied in this study. Three patients were treated with helical tomotherapy (HT), 48 with volumetric modulated arc therapy (VMAT) *via* the RapidArc unit (RA), and 4 with step-and-shoot IMRT (SaS-IMRT). The treatment planning systems were as follows: Hi Art TomoTherapy 2.2.4.1 (Accuray, United States) for the TomoTherapy unit (HT), Varian Eclipse 10.0 for the RA, and Philips Pinnacle 8.0 m for the Elekta Precise Unit (SaS-IMRT, Elekta, Sweden).

### Acute Toxicities and Short-Term Efficacy

**Figure 1:**
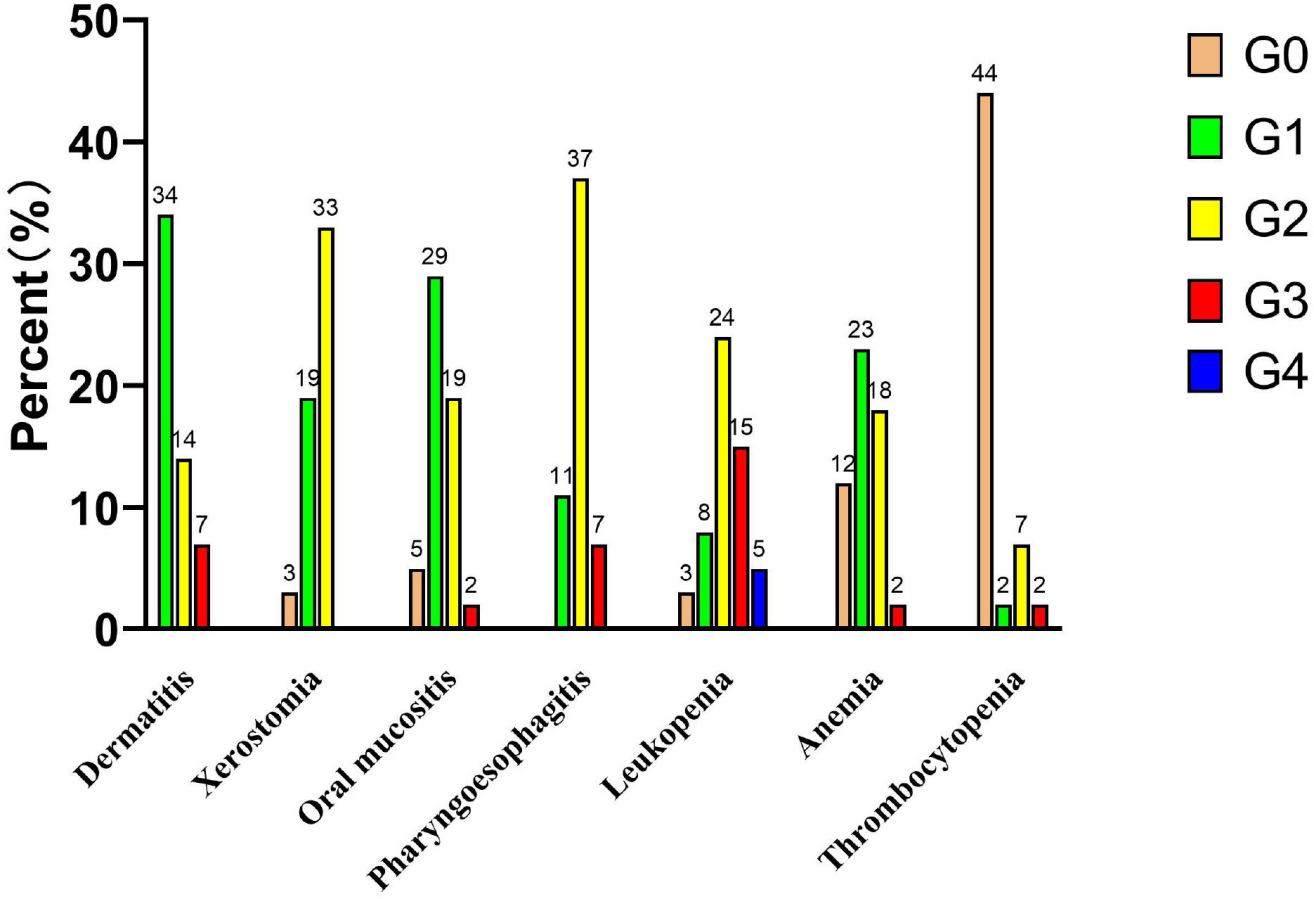
Acute toxicities (*n* = 55).

The incidences of acute dermatitis, xerostomia, oral mucositis, and pharyngoesophagitis are shown in Figure 1. No grade 2 or worse acute xerostomia was noted. Only two patients (T4aN2cM0 and T3N2cM0) developed grade 3 oral mucositis. No grade 4 pharyngoesophagitis was noted. The incidences of grade 3 acute dermatitis, oral mucositis, and pharyngoesophagitis were 12.7% (7/55), 3.6% (2/55), and 12.7% (7/55), respectively. Five patients underwent tracheotomy before CCRT, and two patients underwent tracheotomy due to severe laryngeal toxicity after 29 fractions of radiation therapy and 1 month immediately after radiation therapy, respectively. However, the tracheotomy tube was subsequently removed in these two patients who survived to the end of follow-up.

Evaluation of primary lesions showed complete responses (CR) in 13 (23.6%), partial responses (PR) in 39 (70.9%), and stable diseases (SD) in 3 (5.5%) patients.

### Follow-Up Time

The median follow-up time was 48.0 months (range 5.5–74 months) for all the patients and 65 months (range 41–74 months) for survivors.

### Treatment Outcomes and Functional Larynx Preservation

One patient developed late dysphagia and had a nasogastric tube feeding. Except for two patients who had undergone tracheotomy due to acute laryngeal edema, no patients developed late laryngeal stenosis. The main late toxicity was hoarseness or sore throat, with an incidence of 32.7% (18/55). Three patients developed biopsy-confirmed radiation-related damage, two of whom had local tumor recurrence and died of hemorrhage; the remaining patient was still alive at follow-up. All three patients had hypopharyngeal tumors (stages T3N2bN0, T4aN2cM0, and T2N2bM0). The main cause of failure was local recurrence, which developed in 24 cases (43.6%). The regional recurrence developed was observed in three cases, and one of whom had both local and regional relapse. The lung metastasis occurred in four cases, and the bone metastasis occurred in one case. Two patients developed esophageal cancer during the follow-up.

**Figure 2:**
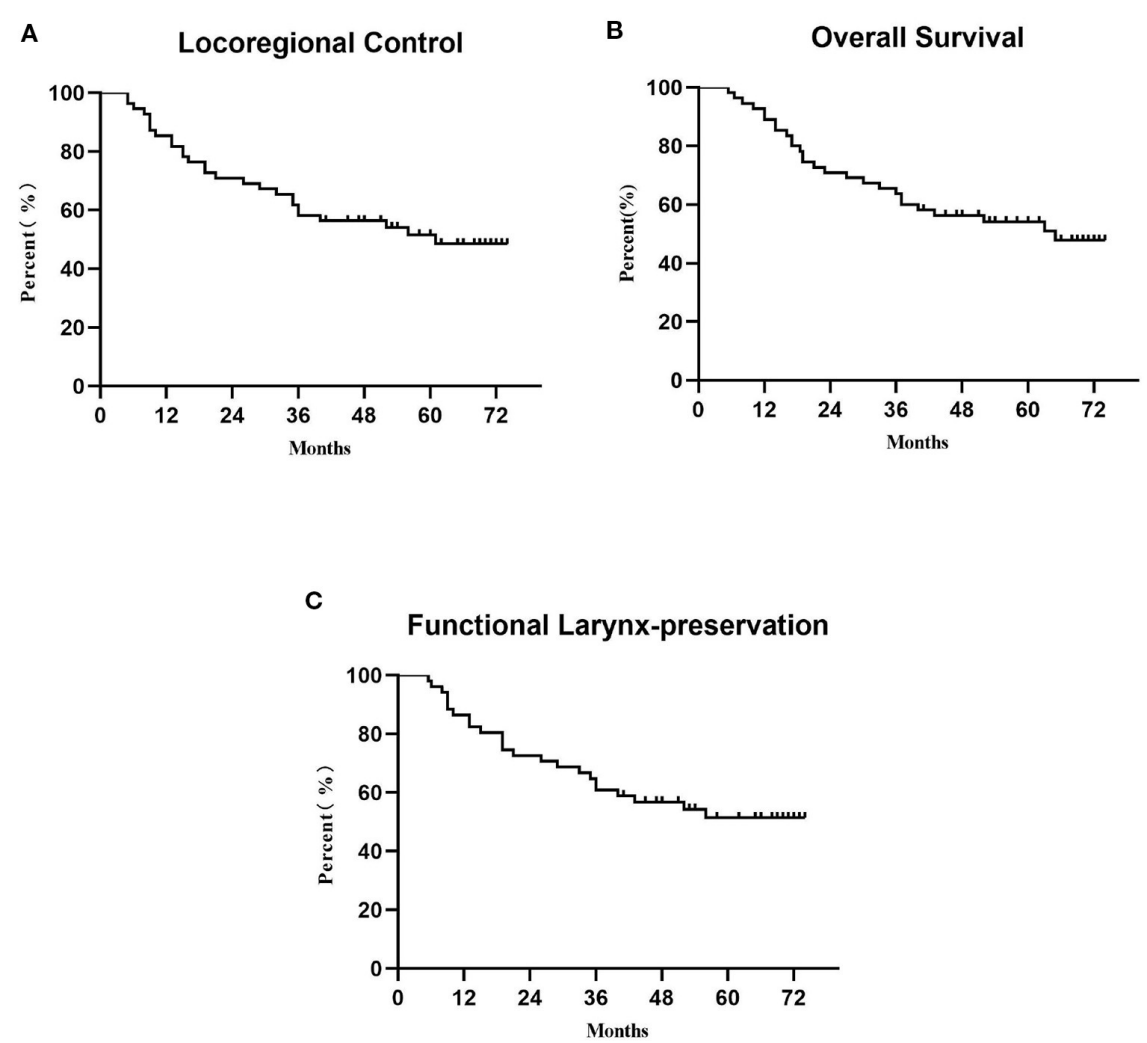
Survival analysis: **(A)** locoregional control, **(B)** overall survival, and **(C)** functional larynx preservation.

**Figure 3:**
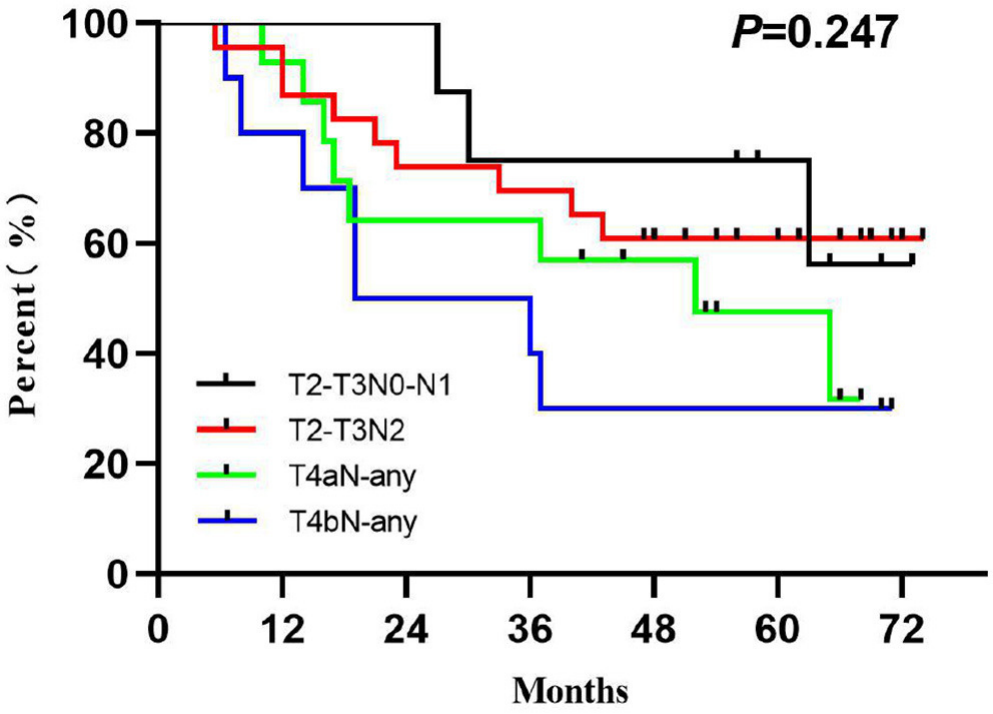
Overall survival by tumor burden.

The LRC and OS were 58.2 and 51.5% for 3 years and 63.6 and 54.1% for 5 years (Figure 2). The 1-, 3-, and 5-year functional larynx-preservation survival were 80.5, 60.9, and 51.5%; respectively (Figure 2). Patients with hypopharyngeal carcinoma had a shorter survival time than those with supraglottic carcinoma, with 5-year OS of 49.6 and 69.2%, respectively, but this difference was not statistical (*p* = 0.297). A log-rank two-sided test showed that there was no independent factor for either LRC or OS. The prognosis of patients with late T stage of tumors was poor, but the statistical evidence was not sufficient. For patients with T3, T4a, and T4b tumors, the 5-year OS was 62.5, 47.6, and 30.0%, respectively (*p* = 0.233). The survival analysis was further evaluated within each staging subgroup based on tumor burden (Table 2). The low nodal burden group had the best 5-year OS, and the very advanced group had the worst (Figure 3). For patients with T4b tumors, the median survival was 19 months, and only three patients were still alive (30%), but the survival of these three patients was longer than 70 months. Patients with severe acute pharyngoesophagitis had a poor prognosis (13), but the statistical evidence was not sufficient after long-term follow-up (*p* = 0.066). The 3-year OS was only 28.6% in patients with grade 3 acute pharyngoesophagitis, compared with 90.9 and 60.2% in those with grade 1 and 2, respectively.

**Table 2:** Survival analysis by tumor and node classification.

	**No**.	**Median survival, month**	**OS, %**		***P***
			**3-year**	**5-year**	
					0.247
T2-T3N0-N1 (non-T4, low nodal burden group)	8	60.5	75.0	75.0	
T2-T3N2 (non-T4, high nodal burden group)	23	51	69.6	60.9	
T4aN-any (T4, high tumor burden group)	14	52	64.3	47.6	
T4bN-any (very advanced group)	10	19	40.0	30.0	

## Discussion

Dermatitis, xerostomia, mucositis, and pharyngoesophagitis are the most common radiation-related acute toxicities in patients with LA-SCCH/L and correspond well with the dose delivered to OARs. Late toxicities were also associated with hypopharyngeal dose in locally advanced head-and-neck cancer in an RTOG analysis (8). Many clinical studies have shown that acute toxicities are common in the standard fractionation regimen of CCRT but are usually tolerable. Huang et al. (17) reported that the rates of treatment-related mucositis (≥grade 2) and pharyngitis (≥grade 3) were higher in the CCRT group. Loimu et al. (11) showed that the fractionation regimen of 2 Gy/F had the most common radiation-related side effects of grade 1–2 dermatitis and mucositis, and medication was needed to control mucosal pain in 64% of patients. The conventional fractionated irradiation was also used in the research by Pala et al. (18), and grade 3/4 acute mucositis was the main radiotherapy-related toxicity and was reported in 32% of patients. Another study in which the fractionation regimen was 2.12 Gy/F showed that grade 2 or worse mucositis occurred in 48% of patients who also experienced grade 2 or worse pharyngitis during treatment (19). A large sample size study with 123 patients, showed that patients could tolerate IMRT by fractionated doses up to 2.11–2.20 Gy, with 2-year LRC, OS, and functional larynx-preservation survival rates of 77, 83, and 74%, respectively (20). Ghi et al. (21) compared ICT followed by CCRT and CCRT alone, with conventional fractionated radiotherapy (2 Gy/F), and the rates of grade 3–4 mucositis and dermatitis were 34.5 and 14%, respectively, with no significant difference observed in the acute toxicity during CCRT between ICT and non-ICT use. Dragan et al. (22) retrospectively analyzed simultaneous integrated boost IMRT in patients with head-and-neck squamous cell carcinoma, with a high-risk PTV dose of 2–2.2 Gy/F for the postoperatively group and 2 Gy/F for the definitive irradiated group. Acute grade 3 toxicities were dysphagia (44%), oral and/or oropharyngeal mucositis (40%), and dermatitis (21%), which were higher than those in our study.

The most severe late postradiotherapy complications in LA-SCCH/L were laryngeal necrosis and necrotizing fasciitis (17). Two dose levels were compared in a sequential cohort Phase I/II study by Miah et al. (12), the incidence of grade 3 toxicities was higher in patients with 67.2 Gy/28 F (2.4 Gy/F) than with 63 Gy/28 F (2.25 Gy/F), 87 and 59% patients confronted acute dysphagia with grade 3, respectively. Five-year follow-up data showed that only two patients in the 2.4 Gy/F group and one patient in 2.25 Gy/F group developed grade 3–4 benign pharyngeal strictures (23). In our study, three patients with hypopharyngeal carcinoma developed laryngeal necrosis or necrotizing fasciitis. The dosimetric analysis showed no local high dose, with GTV Dmax of 72.77 Gy vs. pGTV Dmax of 72.97 Gy, which indicates that laryngeal necrotizing fasciitis might occur occasionally and cannot be predicted by the planning dose parameters. In the previous analysis, we found that patients with severe pharyngoesophagitis had a poor prognosis, and it was an independent factor of 2-year OS (13). After prolonged follow-up, no survival-related factors were detected, and some clinical indicators had certain differences in therapeutic effects that did not reach statistical significance. Objectively, this may be due to an insufficient number of cases. Unlike in the previous studies, the patients in our present study refused laryngectomy at the beginning, and salvage surgical intervention had an impact on the OS analysis (see Table 3 for details).

**Table 3:** Selected literature review on fractionation regimens.

**Reference**	**Fractionation dose of GTV**	**Total dose of GTV**	**Years**	***n***	**Tumor sites**	**Stage**	**ICT**	**CCRT**	**Salvage surgery**	**Acute toxicities**	**Survival**
Huang et al. (17)	2 Gy/F	70 Gy	2003–2007	33	Hypopharynx	II–IVA	No	Yes	Yes	Mucositis (≥grade 2) 39.4% Pharyngitis (≥grade 2) 78.8%	year LRPFS 53% 5-year OS 44%
Loimu et al. (11)	2 Gy/F	66–72 Gy	2001–2007	83	Oropharynx, hypopharynx, and larynx (87%)	III–IVB	No	Yes	Yes	Mucositis (grade 3) 24%	2-year LRC 84% 2-year OS 82%
Ghi et al. (21)	2 Gy/F	70 Gy	2003–2006	60	Oropharynx, oral cavity, and hypopharynx	III–IV	No	Yes	Yes	Mucositis (≥grade 3) 41% Dermatitis (≥grade 3) 15%	3-year OS 46.5%
				61			Yes	Yes		Mucositis (≥grade 3) 34.5% Dermatitis (≥grade 3) 14%	3-year OS 57.5%
Dragan et al. (22)	2 Gy/F	70 Gy	2012–2014	76	Oropharynx, hypopharynx, and larynx	III–IV (79%)	5%	56%	Unknown	Mucositis (≥grade 3) 40% Dysphagia(≥grade 3) 44% Dermatitis (grade 3) 22%	3-year LRC 64% 3-year OS 52%
Miah et al. (12) & Gujral et al. (23)	2.25 Gy/F	63 Gy	2002–2008	29	Hypopharynx and larynx	III–IVB (79.3%)	Yes	Yes	Yes	Dysphagia(grade 3) 59% Mucositis (grade 3) 45% Dermatitis (grade 3) 24% Xerostomia(grade 3) 26%	5-year LRPFS 54% 5-year OS 61.9%
	2.4 Gy/F	67.2 Gy		31		III–IVA	94%	Yes	Yes	Dysphagia(grade 3) 87% Mucositis (grade 3) 45% Dermatitis (grade 3) 23% Xerostomia(grade 3) 10%	5-year LRPFS 62.6% 5-year OS 67.6%
Present study	2.3 Gy/F	69 Gy	2013–2015	55	Hypopharynx and supraglottic larynx	III–IVB	Yes	Yes	No	Mucositis (≥grade 2) 38.2% Mucositis (grade 3) 3.6% Pharyngitis (grade 3) 12.7% Dermatitis (grade 3) 12.7% Xerostomia(grade 3) none	3-year LRC 58.2% 5-year LRC 51.5% 3-year OS 63.6% 5-year OS 54.1%

*Years, years of enrollment; n, numbers of patients; ICT, induction chemotherapy; CCRT, concurrent chemoradiotherapy; LRC, locoregional control; OS, overall survival; LRPFS, locoregional progression free survival; GTV, gross target volume*.

We have focused on the fractionation regimen of CRT in non-operative patients in the previous discussion, but for some patients, CRT does not show an absolute clinical advantage. Su et al. (24) reported long-term survival outcomes in patients with SCCH/L, and there was no significant difference in the 5-year OS in patients who received CRT compared with patients treated with laryngectomy; with respect to T stage, a better 5-year OS in T2 stage (52 vs. 31%, *p* = 0.026) but similar in T4 stage (53 vs. 58%, *p* = 0.534) was observed in the CRT group compared with the surgery group in the univariate analysis. Patel et al. (16) evaluated 8,703 patients with stage III/IV (excluding T1 tumors) laryngeal squamous cell carcinoma from the National Cancer Data Base. For T4N0–N3 tumors, total laryngectomy compared with CRT was associated with improved OS, and the median survival and 5-year OS were 57.5 and 37.8 months, respectively (*p* < 0.0001). Among patients with non-T4, low nodal burden disease, no survival differences were observed between CRT and laryngectomy. Patients with non-T4, high nodal burden disease may benefit from definitive CRT in their opinion. We performed a similar grouping with Patel et al., and the median survival (5-year OS) of patients with non-T4, high nodal burden disease and T4N0, high tumor burden disease was 51.0 and 52.0 months, respectively. The results were similar to laryngectomy reported previously. However, the 5-year functional larynx-preservation survival in our study reached 51.5%.

The use of ICT followed by CCRT remains controversial. ICT followed by radiotherapy showed no advantage in the LRC and larynx preservation compared with CCRT according to the 10-year results of the RTOG 91–11 study (25). Su et al. (24) reported similar results from a comparison between CCRT and ICT followed by CCRT in a larynx-preservation subgroup, but the patients who received a better response after ICT achieved significantly longer PFS and OS. In recent years, with the advancement of IMRT and further clinical analysis, ICT followed by CCRT is a suitable choice for selected patients with LA-SCCH/L who have a high risk for locoregional relapse and distant metastases, with the potential advantage of improving the locoregional and distant control (7, 26), as it has shown better long-term prognosis and has been the primary option for the larynx-preservation treatment in many centers. Ghi et al. (21) reported that ICT followed by CCRT improved the outcome of patients with locally advanced head-and-neck cancer, with higher median OS and 3-year OS than CCRT (54.7 vs. 31.7 months and 57.5 vs. 46.5%, respectively), with similar grade 3–4 non-hematological toxicities and complications. The results of Gujral et al. (23) were quite excellent, with 5-year LRC, OS, and laryngeal-preservation rates were all higher than 60%. Franzese et al. (27) reported the results of ICT plus CCRT with the OS at 3 and 5 years of 83 and 73%, respectively. However, 47% (48/102) of patients had oropharyngeal cancer, and only 10% (10/102) had stage T4 disease in this study; thus, the results may be controversial. ICT has been preferred for locally advanced hypopharyngeal carcinoma in our center since 2011, though ICT plus CCRT and CCRT did not show a significant difference in our preliminary study using conventional fractionated radiotherapy (14). In the present study, ICT was selected to ensure the efficacy and compliance of patients with LA-SCCH/L when treated with SMART since 2013. Even if 76.4% (42/55) of patients had hypopharyngeal carcinoma, and 43.7% (24/55) had a high tumor burden, our study using the ICT plus CCRT showed similarly higher survival rates.

## Conclusions

In conclusion, our preliminary results showed satisfactory survival, acceptable severe toxicities, and a high functional larynx-preservation rate by ICT combined with SMART. Thirty percent (3/10) of patients with T4b tumors had a long-term survival (70–71 months), which affected the data statistics. Because the number of cases was not insufficient, we focus on choosing the appropriate non-operative population in the future.

## Data Availability Statement

The original contributions presented in the study are included in the article/supplementary material, further inquiries can be directed to the corresponding author/s.

## Ethics Statement

All patients signed, at hospital admission, consent for the use of their data for scientific investigation. This subject was reviewed by the Ethics Committee of the Chinese PLA General Hospital.

## Author Contributions

BC and LMa contributed to the conception of the study and performed the data analyses. LMe and JM contributed to the manuscript preparation. BQ, SX, and FL helped to perform the analysis and provided constructive discussions. All authors read and approved the final version of the paper.

## Conflict of Interest

The authors declare that the research was conducted in the absence of any commercial or financial relationships that could be construed as a potential conflict of interest.
